# Circadian activity predicts breeding phenology in the Asian burying beetle *Nicrophorus nepalensis*

**DOI:** 10.1098/rsos.250624

**Published:** 2025-06-18

**Authors:** Hao Chen, Dustin R. Rubenstein, Guan-Shuo Mai, Chung-Fan Chang, Sheng-Feng Shen

**Affiliations:** ^1^ Biodiversity Research Center, Academia Sinica, Taipei City, Taiwan; ^2^ Institute of Ecology and Evolutionary Biology, National Taiwan University, Taipei City, Taiwan; ^3^ Department of Ecology, Evolution & Environmental Biology, Columbia University in the City of New York, New York, USA

**Keywords:** circadian activity, breeding phenology, machine learning, behavioural monitoring, burying beetle

## Abstract

Climate change continues to alter breeding phenology in a range of plant and animal species across the globe. Traditional methods for assessing when organisms reproduce often rely on time-intensive field observations or destructive sampling, creating an urgent need for efficient, non-invasive approaches to assess reproductive timing. Here, we examined three populations of the Asian burying beetle *Nicrophorus nepalensis* from subtropical Okinawa, Japan (500 m) and Taiwan (1100–3200 m) that were reared under contrasting photoperiods in order to develop a predictive framework linking circadian activity to breeding phenology. Using automated activity monitors, we quantified adult circadian rhythms and used machine learning to predict breeding phenology (seasonal versus year-round breeding) from behaviour alone. Our model achieved 95% accuracy under long-day conditions using just three behavioural features. Notably, it maintained 76% accuracy under short-day conditions when both types are reproductively active, revealing persistent behavioural differences between breeding strategies. These results demonstrate how integrating behavioural monitoring with machine learning can provide a rapid, scalable method for tracking population responses to climate change. This approach also offers novel insights into species’ adaptive responses to shifting seasonal cues across different elevational gradients in the beetles’ native range.

## 1. Introduction

In an era of rapid climate change, shifts in breeding phenology have become one of the most conspicuous biological responses to global warming [1]. Such phenological shifts, particularly in reproductive timing, represent a primary way that species synchronize reproduction with optimal environmental conditions. This adaptive timing mechanism integrates multiple environmental cues, from photoperiod, temperature [2] and precipitation [3] to snow cover [4]. Yet, climate change is disrupting the reliability of these environmental signals, triggering widespread shifts in reproductive timing across taxa [5], often leading to phenological mismatch rather than better adaptation [6].

Traditional methods for assessing breeding phenology often present significant challenges. For example, field studies monitoring insect breeding activities along elevation gradients can require extensive resources and prolonged observation periods of up to 19 days per breeding event [7]. Alternative approaches using histological examination of reproductive organs, while precise, are inherently destructive and preclude longitudinal monitoring [8–10]. More importantly, these methods become extremely time-consuming and impractical when research aims to track large sample sizes or conduct long-term monitoring. For example, assessing the reproductive status of hundreds of individuals across multiple time points or locations using traditional methods would require thousands of dissections or breeding experiments. These methodological constraints have limited our ability to effectively track rapid phenological responses to environmental change.

Behavioural rhythms offer a promising alternative indicator of reproductive state. Across diverse taxa, circadian activity patterns show consistent relationships with breeding conditions. For example, polygynous water skinks (*Eulamprus heatwolei*) display increased movement and social interactions during periods of breeding, correlating strongly with reproductive success [11]. Alpine chamois (*Rupicapra rupicapra*) shift from unimodal to multimodal daily activity patterns during the breeding season [12–14], while Arctic-breeding shorebirds exhibit distinct activity signatures during reproduction [15]. These synchronized behavioural adaptations reflect the optimization of reproductive timing across temporal niches [16,17]. Although correlations between behaviour and reproductive state have been established, translating these relationships into practical predictive tools remains a challenge. Simply using correlations between behaviour and reproductive status may not be able to identify subtle patterns or deal with complex, nonlinear relationships. In addition, raw behavioural data often contain noise and variability that can reduce predictive accuracy. Although traditional quantitative methods such as chi-square periodogram analysis effectively assess organismal rhythmicity, they remain insufficient for capturing complex associations between activity patterns and breeding phenology. Indeed, when individuals exhibit arrhythmic activity patterns, more sophisticated time-series feature extraction and machine-learning techniques are required to identify the underlying behavioural signatures. Machine-learning approaches offer unique benefits, including their ability to identify the most predictive features from complex data, effectively model nonlinear relationships and efficiently process large volumes of new data once training is complete [18–20].

Recent advances in machine learning have created new opportunities to leverage these behavioural indicators for reproductive monitoring. While machine learning has successfully characterized mammalian behavioural patterns [21] and emotional states [22], its application to reproductive phenology remains largely unexplored. In this study, we first determine the reproductive phenology (seasonal versus year-round breeding) of different populations through traditional breeding experiments, and then collect detailed behavioural data from these populations that is processed through a machine-learning pipeline. Based on this training dataset, we develop a machine-learning model capable of predicting breeding phenology from behavioural traits alone. This ‘train once, apply many times’ approach can significantly increase the efficiency and scale of subsequent research after the initial training phase, making it particularly suitable for tracking phenological shifts under climate change.

*Nicrophorus* burying beetles are an ideal model organism for this approach due to their strong responsiveness to environmental cues, particularly photoperiod [23]. The Asian burying beetle *Nicrophorus nepalensis* is distributed widely across East Asia, from low-elevation subtropical regions to high-elevation temperate zones [23]. Previous studies have shown that *N. nepalensis* populations from different elevations exhibit markedly different reproductive strategies: high-elevation populations (>3000 m) breed year-round, whereas low-elevation populations exhibit seasonal breeding patterns, reproducing only during specific seasons [23]. This clear geographic and reproductive variation provides an ideal system for investigating the effects of climate change on breeding phenology. Here, we demonstrate how integrating automated activity monitoring with machine learning can reliably predict reproductive seasonality in populations of *N. nepalensis*. By combining high-resolution behavioural data with controlled breeding experiments, we developed a random forest model that can accurately distinguish seasonal from year-round breeders based on circadian activity patterns alone. This non-invasive approach enables unprecedented temporal and spatial resolution in tracking breeding phenology, offering a powerful new tool for monitoring biodiversity responses to environmental change.

## 2. Methods

### 2.1. Study organism

Burying beetles (Silphidae: *Nicrophorus*) utilize small vertebrate carcasses for both reproduction and provisioning offspring [24]. During and after mating, pairs prepare carcasses by removing hair [25] and applying antimicrobial secretions [26]. The processed carcass is then shaped into a brood ball, buried and surrounded by female-laid eggs. After approximately 14 days, third-instar larvae disperse for pupation. Following a 1.5 month pupal period, emerged adults require three to four weeks of feeding to achieve sexual maturity [27] before initiating subsequent breeding cycles.

Despite being stenothermic and cold-adapted [28], *N. nepalensis* maintains a broad distribution across Asia [29]. Populations exhibit distinct breeding seasons across their range, with reproductive behaviour and ovarian development primarily regulated by photoperiodic cues [23]. This interpopulation variation in breeding phenology reflects local adaptation in reproductive photoperiodism [23].

### 2.2. Experimental procedures

To investigate adaptive strategies across environmental gradients, we selected three natural populations of *N. nepalensis* from distinct latitudinal and elevation locations: subtropical lowland Okinawa Island, Japan (26.69° N, <500 m), mid-elevation Mt. Yangming, Taiwan (25.18° N, <1100 m) and high-elevation Mt. Hehuan, Taiwan (24.14° N, <3200 m). Previous research revealed that while the high-elevation Mt. Hehuan population remains active year-round [23], the lower-elevation populations in Okinawa and Mt. Yangming exhibit distinct winter activity peaks (Shen *et al.*, unpublished data).

We collected beetles using hanging pitfall traps baited with decomposing pork. Wild-caught adults were then used exclusively to establish laboratory breeding lines, with all experimental subjects comprising either offspring of wild-caught parents (first laboratory generation) or their descendants from subsequent laboratory generations (F1, F2, etc.). Third-instar larvae were randomly assigned to either short- (10L : 14D) or long-day (14L : 10D) photoperiod treatments throughout pupation (1.5 months pre-emergence) and sexual maturation (1 month post-emergence). Based on previous findings demonstrating that winter-active seasonal breeders maintain their non-reproductive state under long-day conditions [23], all activity measurements and breeding trials were conducted under long-day conditions (14L : 10D).

### 2.3. Locomotor activity measurement

**Figure 1.**
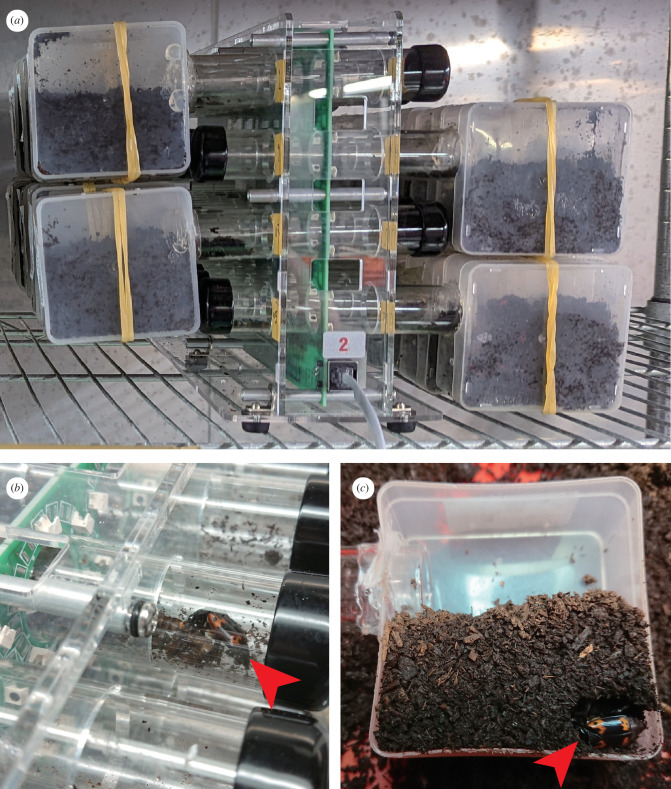
Quantifying circadian activity in *N. nepalensis* using the locomotor activity monitor. (*a*) Monitor array containing 32 channels with transparent glass tubes for simultaneous individual monitoring. (*b*) A food source was provided ad libitum at one end of each tube. (*c*) Opposite ends connected to a 320 ml transparent plastic container filled with soil, serving as refuge. Red arrows indicate beetle positions.

We quantified circadian activity using the Locomotor Activity Monitor 25 system (LAM25, TriKinetics Inc., Waltham, MA, USA) [30]. Each monitor contained 32 channels equipped with transparent glass tubes (PGT 25 × 125 mm, TriKinetics Inc.) (figure 1*a*), surrounded by three pairs of infrared emitter-detector gates. Activity was recorded when beetles interrupted these infrared beams. Fresh superworm *(Zophobas morio*) larvae were provided ad libitum as a food source at one end of each tube (figure 1*b*), with the opposite end connected to a 320 ml transparent plastic container filled with soil for refuge (figure 1*c*). Activity data were transmitted through the Power Supply Interface Unit (PSIU9, TriKinetics Inc.) and collected in 1 min bins using DAM-System3 software (TriKinetics Inc.). Following a 24 h acclimation period, activity was monitored continuously for 63 h under controlled conditions (14L : 10D; 16 ± 3°C; RH: 83–100%) in a walk-in growth chamber, with the dark period beginning at 19.00 to minimize external disturbances.

### 2.4. Breeding type assessment

We assessed reproductive success under different photoperiod treatments (10L : 14D versus 14L : 10D) across all three populations to determine breeding type. Following activity measurements and a minimum 24 h rest period, beetles were paired for breeding trials under standardized conditions (14L : 10D; 16 ± 3°C; RH: 83–100%; Hipoint growth chamber). Each trial used unique male–female pairs in transparent breeding boxes (21 × 13 × 13 cm) containing 10 cm of soil and a 75 ± 7.5 g fresh mouse carcass. After two weeks, breeding success was assessed by the presence of third-instar larvae. Populations successfully breeding under only one photoperiod treatment were classified as seasonal breeders, while those maintaining high reproductive success under both short- and long-day treatments were designated as year-round breeders.

### 2.5. Data analysis

Due to complete separation in breeding trials (where all pairs either succeeded or failed within certain treatments), we used a Bayesian generalized linear model (*bayesglm* function, *arm* R package) with Tukey pairwise comparisons to analyse differences in reproductive success across populations and photoperiod treatments. We then trained a random forest classifier [31] using locomotor activity data (§2.3) as explanatory variables and breeding type classifications (§2.4) as response variables. This approach efficiently handles complex time-series features while avoiding distributional assumptions and multicollinearity issues common to traditional models [19]. We supplemented this analysis with SHapley Additive exPlanations (SHAP) to enhance model explainability.

Activity data were separated into short- and long-day datasets to examine the circadian patterns’ predictive power for breeding type. Raw activity data underwent preprocessing using a sliding window approach and logarithmic transformation to smooth signals and reduce noise [32]. Datasets were randomly split into training (70%) and testing (30%) sets. Using the *tsfresh* Python package [33], we initially extracted nine common features (*extract_features* function with *default_fc_parameter = MinimalFCParameters()*), including sum values, mean, median, minimum, maximum, root mean square, variance, standard deviation and length. Since three features (median, minimum and length) showed no variance across individuals (median and minimum = 0, length = measurement duration), they were excluded from further analysis.

Using the remaining six features, we trained a preliminary (first) random forest classifier and calculated corresponding SHAP values. To optimize model complexity, we performed hierarchical clustering on these features using the *shap.utils.hclust* function, identifying and eliminating redundant features where clustering tree terminals contained multiple highly correlated variables. We selected representative features from each cluster based on SHAP importance rankings, retaining the most influential feature while removing others providing redundant information. This minimal feature set trained our final (second) random forest classifier, maintaining consistent parameters and random states with the preliminary model. For comparison, we trained a third classifier using *tsfresh*’s comprehensive feature set (787 features) to evaluate whether our minimal feature set adequately captured behaviourally relevant patterns for discriminating breeding types.

Finally, we compared feature differences between seasonal and year-round breeders under both photoperiod treatments using general linear models (GLMs) with Tukey pairwise comparisons. Count-based time-series features were analysed using negative binomial regression (*glm.nb* function, *MASS* R package), while continuous features used linear regression. Rhythmicity was determined using chi-square periodogram analysis [34]. Specifically, the activity counts were analysed in the period range of 18–30 h, with a resolution of 0.1 h. Beetles were classified as rhythmic if at least one peak in the period range exceeded the *p* ≤ 0.05 significance level. Individuals lacking a significant peak were classified as arrhythmic. All analyses were performed using Python v. 3.8.8 and R v. 4.3.1.

## 3. Results

**Figure 2.**
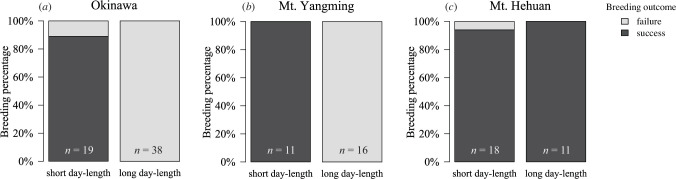
Breeding success of *N. nepalensis* populations in the laboratory that originated in (*a*) Okinawa, Japan (500 m), (*b*) Mt. Yangming, Taiwan (1100 m) and (*c*) Mt. Hehuan, Taiwan (3200 m) under contrasting photoperiod treatments. Stacked bars represent the proportion of successful (dark grey) and failed (light grey) breeding pairs. Sample sizes (*n*) for breeding pairs are indicated at the base of each bar. Breeding success is defined by the successful emergence of third-instar larvae.

Through controlled breeding experiments, we uncovered distinct reproductive strategies among *N. nepalensis* populations from different elevations (Okinawa: 500 m; Mt. Yangming: 1100 m; Mt. Hehuan: 3200 m) that aligned with specific circadian activity patterns. Populations exhibited significant variation in their reproductive responses to photoperiod (Bayesian GLM, population × treatment, $\chi_{1}^{2}$ = 12.36, *p* = 0.002, table 1a). Both low-elevation populations from Okinawa and Mt. Yangming successfully reproduced only when exposed to short-day conditions during development (Okinawa: *p* < 0.001, figure 2*a*; Mt. Yangming: *p* = 0.001, figure 2*b*; table 1b), characterizing them as seasonal breeders. In contrast, the high-elevation Mt. Hehuan population maintained consistently high reproductive success regardless of photoperiod (*p* = 1.00, figure 2*c*, table 1b), exhibiting a year-round breeding strategy.

**Table 1.** Population-specific reproductive responses to developmental photoperiod treatments in laboratory-reared *N. nepalensis*. (a) The effects of population origin, photoperiod treatment and their interaction on breeding success across three populations. (b) Pairwise comparisons of breeding success between individuals developed under short- (10L : 14D) or long-day (14L : 10D) conditions. Significant effects are in bold.

(a)			
variable	*χ^2^*	d.f.	*p*
population	46.19	2	**<0.001**
treatment	79.31	1	**<0.001**
population × treatment	12.36	2	**0.002**
*n* = 113			

(b)				
contrast	odds ratio	s.e.	*Z* ratio	*p*
Okinawa (*n* = 57): short day-length–long day-length	1409.11	2904.5	3.52	**<0.001**
Mt. Yangming (*n* = 27): short day-length–long day-length	2656.02	6442.3	3.25	**0.001**
Mt. Hehuan (*n* = 29): short day-length–long day-length	1.00	1.3	−0.003	1.00

**Figure 3.**
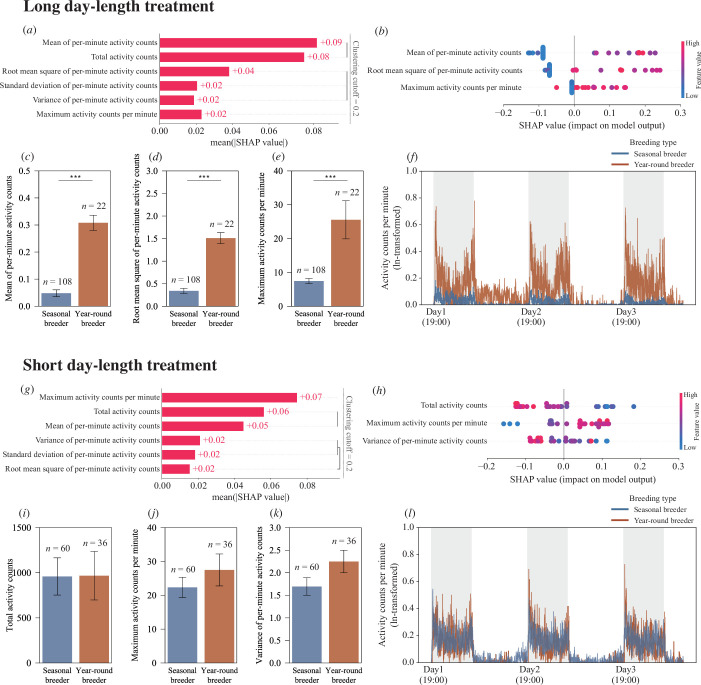
Activity patterns distinguish breeding types differently under long-day versus short-day treatments. (*a–f*) Long-day treatment (14L : 10D): (*a*) SHAP summary plot showing relative importance (mean SHAP value) of activity features in predicting breeding type. Hierarchical clustering tree reveals three distinct feature groups: maximum activity counts per minute provide independent information, while mean of per-minute activity counts and total activity counts form a second correlated cluster, and root mean square, standard deviation and variance of per-minute activity form a third cluster. Representative features with the highest mean SHAP values were selected from each cluster. (*b*) SHAP beeswarm plot illustrating feature contributions to model predictions, where each point represents an individual beetle (red = high values, blue = low values). SHAP values > 0 indicate stronger prediction for year-round breeders, whereas SHAP values < 0 indicate seasonal breeders. (*c–e*) Breeding type comparisons showing: (*c*) mean of per-minute activity; (*d*) root mean square of per-minute activity; and (*e*) maximum activity counts per minute. (*f*) Daily activity patterns under long-day treatment. (*g–l*) Short-day treatment (10L : 14D): (*g*) SHAP summary plot with hierarchical clustering of features. Hierarchical clustering tree reveals three distinct feature groups: maximum activity counts per minute provide independent information, while total activity counts and mean per-minute activity counts form a second correlated cluster, and variance, standard deviation and root mean square of per-minute activity form a third cluster. Representative features with the highest mean SHAP values were selected from each cluster. (*h*) SHAP beeswarm plot illustrating feature contributions to model predictions. (*i–k*) Breeding type comparisons showing: (*i*) total activity counts; (*j*) maximum activity counts per minute; and (*k*) variance of per-minute activity. (*l*) Daily activity patterns under short-day treatment. Sample sizes (*n*) for each group are indicated within panels. Error bars represent standard error. *p* < 0.1, *p < 0.05, **p < 0.01, ***p < 0.001.

To investigate the relationship between circadian activity patterns and breeding strategies, we monitored the locomotor activity of 226 beetles across the three populations (Okinawa: *n* = 114; Mt. Yangming: *n* = 54; Mt. Hehuan: *n* = 58) under long-day conditions (14L : 10D) using our automated activity monitoring system (LAM25). The hierarchical clustering analysis of these behavioural data revealed three distinct clusters (figure 3*a*): one cluster comprising highly correlated measures of mean and total activity counts, another cluster containing root mean square, standard deviation and variance of per-minute activity counts and a third cluster containing only maximum counts per minute. From the first two clusters, we selected mean and root mean square as representative features, as they showed the highest importance rankings within their respective clusters in our preliminary random forest model. Given the complex nature of behavioural data, as well as the potential nonlinear relationships between activity patterns and breeding strategies, we used machine learning to determine how each behavioural feature contributes to the model’s predictions and to ensure biological interpretability.

Using these three representative features—mean, root mean square and maximum counts per minute—we developed a streamlined random forest classifier that achieved exceptional predictive accuracy in distinguishing between seasonal and year-round breeding types (accuracy = 0.949, F1 score = 0.949). This classification accuracy demonstrates that by analysing beetles’ circadian activity patterns alone, we can reliably determine whether they belong to seasonal breeding or year-round breeding populations, without the need for time-consuming breeding experiments or invasive physiological dissections. Remarkably, this minimal feature set achieved comparable performance to a comprehensive model incorporating 787 activity features (accuracy = 0.949, F1 score = 0.949). SHAP analysis revealed that the mean of activity counts per minute contributed most strongly to the model’s predictions, with higher values of these features typically indicating year-round breeders (figure 3*b* and *f*).

The predictive power of activity patterns varied with photoperiod. Under short-day treatments, hierarchical clustering identified three distinct feature groups (figure 3*g*). A random forest model using the representative features from each cluster (maximum counts per minute, total activity counts and variance) achieved moderate accuracy (accuracy = 0.621, F1 score = 0.588). However, when incorporating the comprehensive feature set, model performance improved substantially (accuracy = 0.759, F1 score = 0.756). This marked improvement in classification accuracy reveals that while seasonal and year-round breeders may appear to exhibit similar activity patterns under short-day conditions, their behavioural responses remain fundamentally distinct when analysed at a finer scale. This finding suggests that despite both types being physiologically capable of reproduction under winter conditions (while measured in the activity tubes), they maintain distinct behavioural signatures that reflect their differing life-history strategies. SHAP analysis revealed an unexpected pattern: while higher maximum activity still predicted year-round breeders, higher total activity was associated with seasonal breeders (figure 3*h*), potentially reflecting their sustained daytime activity (figure 3*l*).

Traditional statistical analyses (GLM) confirmed significant behavioural differences between breeding types across photoperiod treatments (treatment × breeding type, *p* ≤ 0.001, electronic supplementary material, tables S1a–5a). Under long-day treatments, year-round breeders exhibited significantly higher mean (*p* < 0.001, figure 3*c*, electronic supplementary material, table S1b), root mean square (*p* < 0.001, figure 3*d*, electronic supplementary material, table S2b) and maximum counts per minute (*p* < 0.001, figure 3*e*, electronic supplementary material, table S3b) than seasonal breeders. However, under short-day treatments, both breeding types showed comparable levels across all three representative features (total activity: *p* = 0.98, figure 3*i*, electronic supplementary material, table S4b; maximum counts per minute: *p* = 0.34, figure 3*j*, electronic supplementary material, table S3b; variance: *p* = 0.08, figure 3*k*, electronic supplementary material, table S5b).

Finally, we also assessed individual rhythmicity under different photoperiod treatments using chi-square periodogram analysis. Under the short-day treatment, both seasonal (55%) and year-round breeders (55.6%) showed similar proportions of rhythmic individuals (*p* = 0.96, electronic supplementary material, table S6b). However, under the long-day treatment, seasonal breeders displayed notably low rhythmicity, with only 17.6% of individuals exhibiting rhythmic patterns, while year-round breeders maintained high rhythmicity (68.2%) (*p* < 0.001, electronic supplementary material, figure S1 and table S6b).

## 4. Discussion

**Figure 4.**
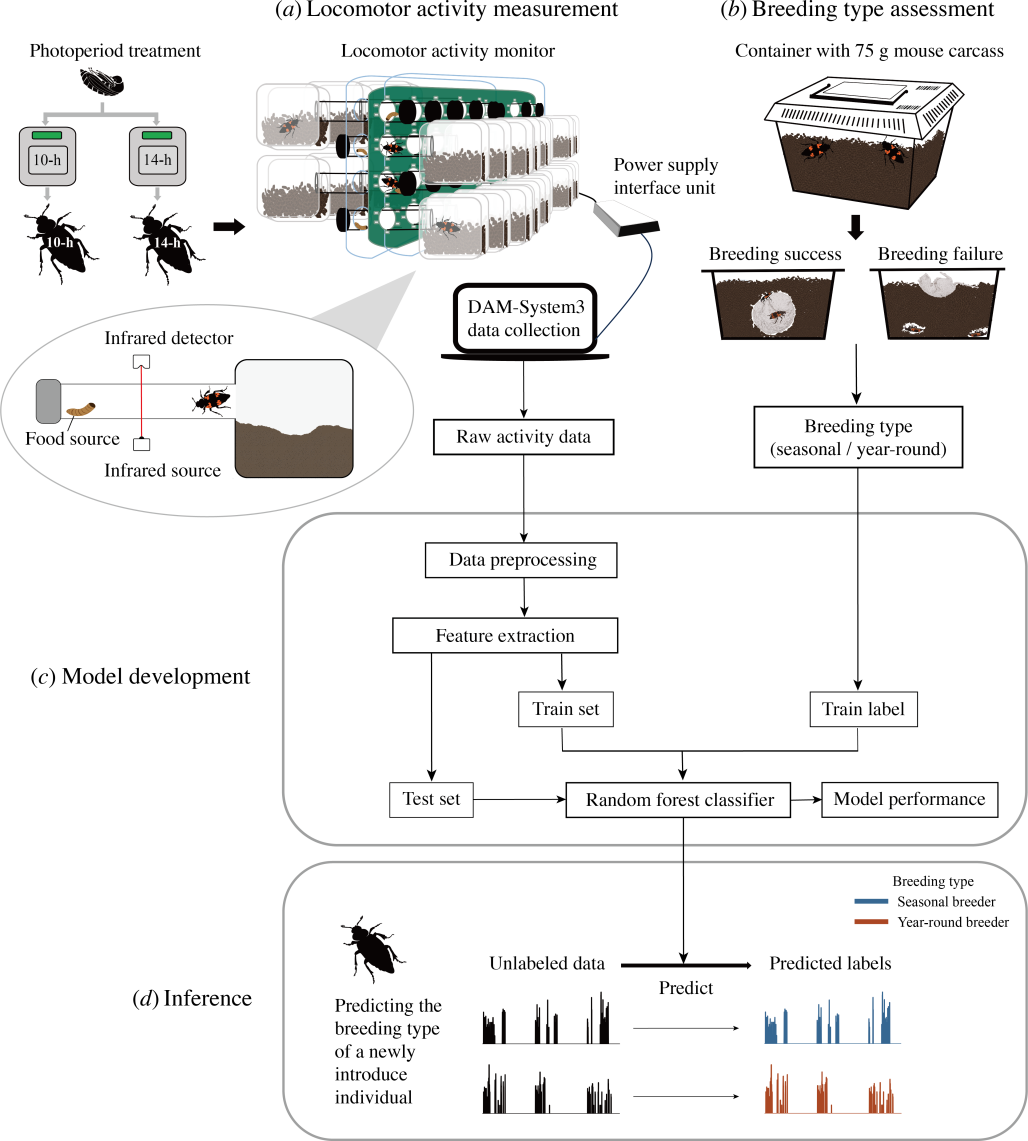
Framework for predicting insect breeding seasonality using circadian activity patterns. Prior to activity monitoring, individuals were randomly assigned to either short- (10L : 14D) or long-day (14L : 10D) photoperiod treatments throughout pupation and sexual maturation. (*a*) Locomotor activity measurement: beetles exposed to different photoperiod treatments are monitored using infrared detectors to record daily activity patterns. Inset shows the experimental set-up with food source and shelter area. (*b*) Breeding type assessment using standardized breeding trials with a 75 g mouse carcass to classify individuals as seasonal or year-round breeders. (*c*) Model development pipeline showing data processing steps from raw activity data through random forest classifier training. (*d*) Model application demonstrating how unlabelled activity patterns from newly introduced individuals can be classified into breeding types, enabling rapid assessment of reproductive seasonality. This non-invasive approach allows efficient monitoring of phenological responses to environmental change.

This study demonstrates how behavioural monitoring can provide a robust non-invasive method for predicting insect breeding phenology (figure 4). By analysing *N. nepalensis* circadian activity patterns with machine learning, we achieved 94.9% accuracy in distinguishing seasonal from year-round breeders. Even more remarkably, our comprehensive behavioural analysis revealed that these populations maintain distinct activity signatures under short-day conditions (75.9% accuracy), despite both types being reproductively active.

The main value of this study lies in establishing an alternative assessment method that, after an initial training phase, can significantly improve the efficiency of large-scale phenological assessments. Although our method requires up-front investment to establish the relationship between circadian activity and breeding phenology (we used activity data from 226 individuals and corresponding breeding experiments), once established, the model can predict the reproductive status of hundreds or thousands of individuals without time-consuming breeding experiments or invasive physiological dissections. This approach is particularly suited for research projects requiring long-term monitoring or large-scale assessment, such as tracking phenological responses to climate change, or studying differences in breeding timing across elevational and latitudinal gradients. This methodological breakthrough addresses a critical challenge in studying phenological responses to climate change, as traditional approaches to assessing reproductive status typically require intensive fieldwork [7] or destructive sampling [8,9]. The strong association between circadian activity and reproductive state aligns with growing evidence that behavioural rhythms serve as reliable indicators of reproductive status across taxa. The applicability extends beyond reproductive phenology to other traits related to circadian activity, such as migration timing [35] and hibernating diapause [36].

Particularly noteworthy is not only the distinct activity level divergence between seasonal and year-round breeders under long-day conditions, but also their persistent behavioural differences under short-day conditions when both types are reproductively active. These behavioural signatures probably reflect deeper adaptations to local temporal niches beyond reproductive timing, suggesting that populations have evolved integrated life-history strategies manifesting in their activity patterns regardless of current reproductive state [37]. This finding extends our understanding of how animals optimize temporal activity patterns to maximize reproductive success, a key aspect of life-history evolution [38]. Circadian rhythms present in species are associated with anticipation of environmental changes and appropriate timing of specific responses [16,39]. For instance, many animals exhibit predictable daily fluctuations in predation risk and have evolved activity patterns that maximize foraging while minimizing mortality risk [40]. Such temporal adaptations are documented across diverse ecosystems, from aquatic fish [41] to desert rodents [42] and cave-dwelling organisms like harvestman [43], demonstrating fine-scale partitioning of temporal niches.

Traditional methods for assessing insect reproductive status have significant limitations. Histological analyses, while accurate, are time-consuming and largely restricted to female reproductive organ assessment. As demonstrated in studies of fruit flies [10] and burying beetles [23], determining female ovarian development stages typically requires dissection and detailed histological analysis. Even seemingly more straightforward methods, such as the gonadosomatic index (GSI), are dependent on precise sampling timing and frequently fail to accurately capture the dynamics of breeding seasons [44]. Our chi-square periodogram analysis further revealed a difference in rhythmicity between breeding types under long-day conditions, with seasonal breeders showing significantly reduced rhythmicity (17.6%) compared with year-round breeders (68.2%). This finding highlights the complex relationship between circadian rhythmicity and reproductive status and demonstrates how machine-learning approaches can extract valuable information from even seemingly arrhythmic activity patterns. This non-invasive method simultaneously monitors both male and female reproductive behaviour, eliminating gender bias inherent in traditional approaches. Furthermore, automated activity recording systems provide unprecedented temporal resolution through continuous data collection, far surpassing traditional field surveys or laboratory dissections. Behavioural monitoring enables efficient tracking of reproductive state changes, particularly suitable for species with distinct reproductive behaviours like burying beetles, making our method valuable for studying reproductive responses to photoperiod and temperature changes, as well as assessing climate change impacts.

As climate change increasingly disrupts seasonal environmental cues [5], understanding and predicting shifts in breeding phenology becomes crucial for conservation. Our method demonstrates practical value in several application scenarios: in time-series phenological monitoring, researchers could track seasonal changes in breeding status without continuous breeding experiments [7]; in cross-annual comparisons, subtle changes in breeding timing between years can be tracked, particularly in response to gradual climatic changes [45]; in elevational gradient studies, deploying activity monitors at multiple elevations could simultaneously reveal breeding status under different ecological conditions without large-scale breeding experiments at each site [46]. Our machine-learning approach, based on easily observable behavioural patterns, provides a scalable tool for monitoring phenological responses across populations and species. This is particularly valuable for tracking rapid evolutionary responses to environmental change, as behavioural adaptations often precede morphological or physiological changes. Although initially requiring laboratory validation, this approach could be adapted for field applications through portable activity monitors or other methods for collecting circadian activity data without extended laboratory housing [47]. The efficiency and non-invasive nature of automated behavioural monitoring integrated with machine learning makes it ideal for long-term phenological studies across altitudinal and latitudinal gradients [23], potentially revealing how different populations adjust their reproductive timing in response to environmental pressures. Such insights are crucial for predicting species adaptations to future climate scenarios and developing effective conservation strategies [48].

## Data Availability

The data are available in Figshare [49]. Supplementary material is available online [50].
